# Are dementia with Lewy bodies and Parkinson’s disease dementia the same disease?

**DOI:** 10.1186/s12916-018-1016-8

**Published:** 2018-03-06

**Authors:** Kurt A. Jellinger, Amos D. Korczyn

**Affiliations:** 1 Institute of Clinical Neurobiology, Alberichgasse 5/13, A-1150 Vienna, Austria; 2 Tel-Aviv University, Sackler Faculty of Medicine, Ramat Aviv, Israel

**Keywords:** Dementia with Lewy bodies, Parkinson’s disease dementia, Clinical features, Diagnostic criteria, Neuropathology, Diagnostic tests, Management, Synucleinopathies

## Abstract

**Background:**

Dementia with Lewy bodies (DLB) and Parkinson’s disease dementia (PDD), which share many clinical, neurochemical, and morphological features, have been incorporated into DSM-5 as two separate entities of major neurocognitive disorders with Lewy bodies. Despite clinical overlap, their diagnosis is based on an arbitrary distinction concerning the time of onset of motor and cognitive symptoms, namely as early cognitive impairment in DLB and later onset following that of motor symptoms in PDD. Their morphological hallmarks – cortical and subcortical α-synuclein/Lewy body plus β-amyloid and tau pathologies – are similar, but clinical differences at onset suggest some dissimilar profiles. Based on recent publications, including the fourth consensus report of the DLB Consortium, a critical overview is provided herein.

**Discussion:**

The clinical constellations of DLB and PDD include cognitive impairment, parkinsonism, visual hallucinations, and fluctuating attention. Intravitam PET and postmortem studies have revealed a more pronounced cortical atrophy, elevated cortical and limbic Lewy body pathologies, higher Aβ and tau loads in cortex and striatum in DLB compared to PDD, and earlier cognitive defects in DLB. Conversely, multitracer PET studies have shown no differences in cortical and striatal cholinergic and dopaminergic deficits. Clinical management of both DLB and PDD includes cholinesterase inhibitors and other pharmacologic and non-drug strategies, yet with only mild symptomatic effects. Currently, no disease-modifying therapies are available.

**Conclusion:**

DLB and PDD are important dementia syndromes that overlap in many clinical features, genetics, neuropathology, and management. They are currently considered as subtypes of an α-synuclein-associated disease spectrum (Lewy body diseases), from incidental Lewy body disease and non-demented Parkinson’s disease to PDD, DLB, and DLB with Alzheimer’s disease at the most severe end. Cognitive impairment in these disorders is induced not only by α-synuclein-related neurodegeneration but by multiple regional pathological scores. Both DLB and PDD show heterogeneous pathology and neurochemistry, suggesting that they share important common underlying molecular pathogenesis with Alzheimer’s disease and other proteinopathies. While we prefer to view DLB and PDD as extremes on a continuum, there remains a pressing need to more clearly differentiate these syndromes and to understand the synucleinopathy processes leading to either one.

## Background

The nosologic relationship, as defined by DSM-5 [1, 2], between dementia with Lewy bodies (DLB) and Parkinson’s disease dementia (PDD), both of which are major neurocognitive disorders with α-synuclein (αSyn) deposition/Lewy bodies (LB), is continuously being debated [3–22].

The clinical features of DLB and PDD are similar and include dementia, cognitive fluctuations, and (visual) hallucinations in the setting of clinical or latent parkinsonism. The cognitive domains of both disorders overlap, with progressive executive dysfunctions, visual-spatial abnormalities, and memory disorders [10]. Based on international consensus, DLB is diagnosed when cognitive impairment precedes parkinsonian motor signs or begins within 1 year from its onset [23], whereas in PDD, cognitive impairment develops in the setting of well-established Parkinson’s disease (PD) [24]. DLB patients will also develop parkinsonism of increasing severity over the years, although 25% of them never develop parkinsonian symptoms [25]. Despite different temporal sequences of motor and cognitive deficits and several quantitative clinical differences, both disorders show largely convergent, albeit locally and quantitatively divergent neuropathological lesions, associated with increased Aβ and tau loads in DLB [9, 26–30]. The overlap of clinical and morphological features has led to the debate of whether DLB and PDD are the same disease [17], different phenotypic expressions of the same αSyn/Lewy body disease (LBD) spectrum, or distinct ‘diseases’ [3, 31] sharing genetic risk features with PD and Alzheimer’s disease (AD) [10, 32], despite recent studies indicating a regional overlap of pathologies [33–37]. The present paper will critically review the major current findings in DLB and PDD, their possible nosologic interrelations, and the available biological markers and therapies. Of note, this review does not include mild cognitive impairment in LBD (see [8, 38–46]).

### Clinical features and diagnostic criteria of DLB

The presenting features of DLB can be broadly placed into three categories, namely cognitive impairment, behavioral/psychiatric phenomena, and physical symptoms [47]. Essential for its diagnosis are dementia with moderate memory impairment, deficits in attention, executive dysfunction and visuoperceptual ability, fluctuating cognition (presumably related to thalamic damage and cholinergic imbalance [48]), and recurrent visual hallucinations that are well formed and detailed [2]. Hallucinations in DLB may occur spontaneously, independent of visuospatial and perceptional impairment [49], and possibly related to LBs in the temporal lobe [50], while in PDD they typically occur after dopaminergic therapy [10, 23, 51]. Nevertheless, hallucinations had been reported prior to the levodopa era [52] as well as in drug-naive PD patients even in the premotor phase [53]. Language impairment tends to be mild, with verbal and semantic fluency deficits. Spontaneous parkinsonian features, such as bradykinesia and rigidity, are common in DLB (over 85%) [31], while rest tremor is less frequent [54]. REM sleep behavior disorder (RBD), which shows a high prevalence in DLB and may precede cognitive decline by decades, is now included as a core clinical feature [55]. RBD may reflect a distinct subtype of DLB with earlier disease onset [56], associated with severe brain metabolic decreases [57]; however, as an early manifestation, it is not specific to DLB [58, 59]. The pattern of initial cognitive dysfunction differs between DLB and PDD [60], with greater deficiencies of attention, executive function, and constructive abilities, as well as significantly lower ratings in episodic verbal memory tasks, in DLB [61, 62]. Further, the rate of cognitive decline is reportedly faster in DLB than in PDD and AD [63, 64] (Table 1).

**Table 1:** Clinical overlap and dissimilarities between dementia with Lewy bodies (DLB) and Parkinson disease with dementia (PDD)

Overlap	Dissimilarities
Rigidity, akinesia Cognitive impairments Frontal executive dysfunction Visual-constructive impairment Mild language impairment Mood disturbances (depression, anxiety) REM sleep behavior disorder (RBD) Neuroleptic sensitivity	Some cognitive dysfunctions: deficiencies of attention greater, episodic verbal memory tasks lower in DLB Tremor less frequent in DLB Motor performance: slower walk and poorer balance in DLB Hallucinations (visual) more frequent in DLB Relative timing of dementia and parkinsonism (one year rule) Onset of dementia earlier in PDD Orthostatic hypotension more frequent in DLB Frontal/temporal-associated cognitive subsets more severe in DLB, cognitive decline is faster in DLB/DLB+AD Delusions, visual hallucinations, and attentional fluctuation more frequent in DLB Visual hallucinations: spontaneous in DLB; after L-dopa therapy in PDD, but also in drug-naive cases

*AD* Alzheimer disease

Supporting clinical features for the diagnosis of probable or possible DLB are repeated falls, syncopes, hyposmia, severe autonomic dysfunction, hypersomnia, hallucinations in non-visual modalities, apathy, depression, and severe sensitivity to antipsychotic agents [2, 65]. However, since these changes also occur in advanced PD, they cannot differentiate DLB from PDD, e.g., the prevalence of neuroleptic sensitivity does not differ significantly between them [66].

A diagnosis of clinically probable DLB requires (1) two or more core clinical features to be present, with or without indicative biomarkers, or (2) the presence of only one core clinical feature but with one or more indicative biomarkers [2]. Although the diagnostic specificity of these criteria is high (range 79–100%), the sensitivity can be low (12–88%), improving with additional supporting features such as biomarkers [67–70]. A recent meta-analysis reported a pooled sensitivity, specificity, and accuracy of 60.2% (95% CI 30.9–83.7%), 93.8% (83.8–97.6%), and 79.7% (62.6–90.7%), respectively, for the diagnostic [23] criteria of DLB [68]. Thus, currently, approximately 20% of DLB diagnoses are incorrect [68, 69].

### Clinical features and diagnostic guidelines of PDD

The clinical features of PDD are in many respects similar to those seen in DLB, although, by definition [23, 71], the occurrence of parkinsonism distinguishes one from the other. Rigidity and akinesia occur both in PDD and DLB [62]. Cognitive impairments in PDD are common and are similar in quality to those of DLB [8]. However, the timing, profile, and rate of cognitive decline vary widely; indeed, the average time to dementia after PD diagnosis is almost 10 years, but may be as long as 20 years [39]. Consensus criteria for PDD [24, 72, 73] require cognitive impairment across multiple domains, mood disturbances, and visual-spatial impairment similar to that seen in DLB. Attentional fluctuations, which are characteristic of DLB, are less frequent in PDD [72] but are clinically indistinguishable in the two conditions [74]. Executive functions are probably more impaired in PDD, while language deficits are rare [71]. Visual symptoms, common in PDD [75] likely due to a reduced metabolism in both dorsal and ventral visual pathways [76], include visual hallucinations, although they are less common than in DLB [77]; yet, the phenomenology of hallucinations is similar in both disorders [78]. Other non-motor features, including autonomic dysfunctions and sleep disorders, may occur disproportionally to the severity of dementia [24, 72], while mood disturbances have a similar frequency as in DLB. The psychosis spectrum of PD has recently been reviewed [79]. RBD can evolve in PDD and DLB [80] in up to 90% of patients after > 10 years [81]. Finally, clinical validation efforts for PDD have shown variable diagnostic sensitivity and specificity [82, 83] and should be considered using the Movement Disorder Society criteria for the diagnosis of PDD [84].

### Epidemiology and natural history of DLB and PDD

Approximately 1–2% of those aged above 65 years are diagnosed with DLB worldwide [16], affecting approximately 5% of all dementia cases in those over the age of 75 [85]. Its incidence is 0.7–1.4 new cases/100,000 person-years [16] or 3.5/100,000 person-years [86]. For PDD, the cumulative prevalence is of 75% of PD patients surviving more than 10 years [87], 83% after 20 years [88], and up to 95% by age 90 years [16], with an overall prevalence of 31.1% (95% CI 20.1–42.1) and incidence rates from 0.43 to 1.13/100,000 person-years [89], indicating that, annually, approximately 10% of a PD population will develop dementia [24]. The data concerning age at disease or dementia onset are highly variable. Whereas in the Olmsted County study [86] DLB patients were younger at symptom onset than those with PDD and had more hallucinations and cognitive fluctuations, others have reported younger age at disease onset in PDD [27, 90, 91], or no essential differences between disorders [14, 37, 92, 93].

Individuals with DLB or PDD have an increased mortality compared with the general population [94]. DLB patients with a cerebrospinal fluid (CSF) AD profile and structural MRI changes (hippocampal atrophy) have a shorter survival [95, 96]; similarly, dementia and/or neuritic AD pathology in PD are related to a significantly shorter survival [97]. PDD is associated with high mortality, advancing death by approximately 4 years [98]. For typical DLB, the average survival time from the beginning of symptoms is 5–8 years [99], while rapidly progressing cases have a mean duration of 9 months [100]. In both disorders, older age, hallucinations, and fluctuating dementia at onset are the best predictors of poor outcome [101, 102].

### Diagnostic tests (Table 2)

#### Neuroimaging

The neuroimaging characteristics have been reviewed in a quest for multimodal methods able to improve ante mortem diagnosis [103, 104]. Studies using ^123^I-β-CIT (DaTScan) SPECT or ^18^Fluorodopa PET demonstrated reduced dopamine transport binding in caudate and posterior putamen in DLB compared to AD, but observed no differences between DLB and PDD [105, 106]. Further, lower ^123^I–ioflupane-CIT has been observed in caudate nucleus in DLB and a greater asymmetry of uptake was seen in the posterior putamen in PDD [104, 107]. Dopamine uptake in striatum is significantly lower in PDD compared to DLB (*P* < 0.04), consistent with dopaminergic cell loss in substantia nigra pars compacta and the severity of parkinsonism [108]. The disruption of dopaminergic pathways impacts the modulation of intrinsic brain networks, resulting in poor motor and cognitive performance [109].

**Table 2:** Laboratory findings overlap and dissimilarities between dementia with Lewy bodies (DLB) and Parkinson disease-dementia (PDD)

Overlap	Dissimilarities
Decreased DAT binding in putamen Reduced cardiac MIBG binding Medial temporal lobe relative preservation Occipital hypoperfusion Similar EEG abnormalities Similar metabolic decrease in cerebral cortex GBA mutations	Grey matter cortical atrophy more frequent and more severe in DLB White matter hyperintensities in temporal lobe more severe and more frequent in DLB Different functional connectivity, corticostriatal disruption:PDD: frontal cortical disruption; DLB: parietal and occipital disruption Greater amyloid binding in DLB Tau-PET imaging more severe in DLB Several genetic differences (APOE ε4, TFAM)) Decreased DAT binding in caudate related to functional impairment in DLB, not in PDD SN sonography (size, asymmetry) CSF AD profile more common in DLB CSF αSyn oligomeres increased in PDD

*AD* Alzheimer disease, *DAT* dopamine transporter, *MIBG* scintigraphy using metaiodobenzylguanidine labeled to Iodine-123 or Iodine-131, *SN* substantia nigra, *CSF* cerebrospinal fluid

SPECT imaging using ^123^I–metaiodobenzylguanidine, a marker of postganglionic sympathetic innervation, showed reduced cardiac uptake in both DLB and PDD as compared with AD [110, 111]. The sensitivity, specificity, and accuracy for the diagnosis of probable DLB is 82.4%, 96.3%, and 92.5%, respectively [112]; yet, although specific data on PDD are not available, ^123^I–metaiodobenzylguanidine imaging is unlikely to differentiate PDD from DLB.

Voxel-based morphometric MRI studies revealed greater grey matter loss in frontotemporal, occipital, and parietal areas in DLB compared to PDD [113–118]. Decreased grey matter volumes in association areas (left precuneus and inferior temporal lobe) are correlated with visual hallucinations in DLB [119], and atrophy of caudate, putamen, and pallidum have been observed in DLB but not in PDD [120–123]. However, since greater volume loss in various brain regions has not been statistically confirmed [124], these differences cannot be used for individual diagnoses.

White matter hyperintensities (WMH) on T2-weighted MRI have been observed in parieto-occipital areas in PDD cases with low CSF Aβ levels [125], without significant difference of progression between PDD and DLB [126], but more severe WMHs have been observed in the temporal lobe in DLB [127]. Thus, evaluation of WMH and medial temporal lobe atrophy using MRI may be a powerful diagnostic tool to investigate the progression of AD-related pathology in DLB and perhaps to distinguish DLB from PDD [126, 128]. Magnetic resonance spectroscopy studies found relatively normal N-acetylaspartate/creatinine ratios in DLB, with similar reductions being observed in PDD and AD [129].

PET, perfusion SPECT, and arterial spin labelling MRI studies showed parietal, frontal, temporal, and occipital hypoperfusion common to both entities [104, 130–135]. Further, ^11^C PIB-PET imaging showed increased Aβ brain deposition in more than 50% of DLB cases, with more modest and less frequent Aβ accumulation in PDD [106, 136–139], while others reported increased cortical Aβ binding without dissimilarity between PDD and DLB [140]. Tau-PET imaging, along with temporal atrophy, may indicate co-existing AD pathology in DLB with variable cortical tau ^18^F–AV-1451 uptake, which appears more common than in PDD [141, 142]. Preliminary tau-PET studies suggest a gradient of tau binding from PD/non-demented (minimal) to PDD (low), DLB (intermediate), and AD (highest) [143]. Finally, the recently described additional ^18^F–AV-1451 binding to (neuro)melanin [144] deserves further investigation.

#### Electrophysiology and other studies

EEG abnormalities from posterior leads have been observed in all DLB cases and in three-quarters of those with PDD [145]. Further, a multicenter study supported the validity of quantitative EEG analysis as a tool for diagnosis of both disorders and their distinction from AD [146, 147], although some components may be reduced more in PDD than in DLB [148]. Finally, transcranial sonographic hyperechogenicity was inconclusive in differentiating DLB from PDD [149]; a comparative electro-oculographic study showed similar impairment in all tasks in both disorders [150].

### Genetics

Both DLB and PD are primarily sporadic diseases, yet genetic factors may be involved in their causation. Recent studies have uncovered certain genetic differences between PDD and DLB, albeit none of which is diagnostic. There is a substantial genetic contribution to DLB, heritability being estimated at about 36% [151, 152], while different genetic markers within the α-Syn gene (*SNCA*) may be associated with PDD [153, 154], although this is not unexpected in PD (Table 3). Analyses of *SNCA* expression in PDD and DLB showed an overlap of αSyn biology, indicating that they have distinct genetic etiologies and predicting that several mechanisms may be specific [154]. Genome-wide association studies (GWAS) identified variants in the *GBA*, *SNCA*, *APOE*, and *MAPT* loci influencing the individual risk for DLB, suggesting that it has shared genetic risk features with PD and AD [32, 155], while the *APOE4* haplotype may be an indication of PDD [156]. However, to date, the genetic differences between both entities have not been studied in detail [157]; further studies will increase our understanding of the pathophysiology of these diseases [158].

**Table 3:** Potential genetic risk factors for dementia with Lewy bodies (DLB) and Parkinson’s disease dementia (PDD)

Gene	DLB	PDD
*GBA* (glucocerebrosidase)	Mutations are most prevalent risk factors for sporadic DLB [271, 272]; associated with increased levels of AD pathology [183, 273].	Mutations associated with risk of PDD and agressive cognitive decline [274–283].
*MAPT* (microtubule-associated protein tau) H1 haplotype	Associated with increased risk of DLB [284].	Strongly associated with dementia in PD [153, 275, 285–289].
*APOE* (apolipoprotein E)	*APOE ε4* is overrepresented in DLB, and it is an increased risk for DLB [35, 290].	Mixed evidence for dementia risk in PD [291–297].
*SNCA* (α-synuclein)	Multiplication is not a common cause of DLB [298, 299]	Rare multiplications and mutations are associated with dementia in monogenic PD [292, 300], but show phenotypic variations and clinical heterogeneity [301–306].
*COMT* (catechol-O-methyltransferase)		No evidence for dementia risk [287, 288, 307–309], but polymorphisms may contribute to cognitive deficits in PD [310].
*UBQLN1* (ubiquilin-1) and *FMR1* (fragile X mental retardation protein 1)	No association with cognitive impairment [311, 312].	
*LRRK2* (leucine-rich repeat serine/threonine-protein kinase 2)	Not essential for DLB [313].	No association with PDD [314–322].
*C9orf72* repeat expansion	Not related with DLB [313].	
*RAB39B* (Ras-related protein Rab-39B) mutations	Not related with DLB [323].	

AD: Alzheimer’s disease, PD: Parkinson’s disease

### Fluid biomarkers

The development of broadly applicable CSF and other biomarkers for both DLB and PDD remains elusive, with only few biomarker candidates having been shown to specifically reflect the underlying disease process [159–161] (Table 2). A CSF AD profile is more common in DLB [162], while cortical atrophy in PDD is associated with increased total CSF αSyn and t-tau [159]. However, cognitive impairment in *GBA*-associated PD does not seem to be associated with Aβ and tau profiles in CSF [163]. The elevated tau/Aβ42 index in the order PD < PDD < DLB < AD may be related to an increased AD pathology [164]. Further, levels of αSyn oligomers in CSF are increased in PDD but not in DLB [165–167]. Although many CSF and some plasma markers have been identified in both disorders, very few studies have examined samples from both disorders simultaneously, and only a minority have been confirmed by post mortem studies [167, 168].

### Neuropathology

The pathological substrates of DLB and PDD have been extensively investigated [9, 27, 29, 30, 35–37, 41, 169–181]. The most difficult problem in defining DLB and PDD at autopsy is their relationship with AD. DLB is, in part, conceived as a variant of AD (‘Lewy body variant of AD’) [182] and significant AD pathology is a consistent but not universal finding in both disorders [181]. Cerebral neurofibrillary tangle burden, along with αSyn and Aβ plaque pathology, are the strongest predictors of a shorter interval between motor and dementia symptom onset and shorter survival [183].

The pathological substrate of PDD includes (1) Lewy/αSyn pathology in cortical, limbic, and brainstem structures, (2) AD-related pathologies, and (3) a combination of these lesions that has been shown to most robustly correlate with the severity of cognitive impairment [41, 169, 173, 174]. Approximately 50% of PDD patients showed Braak LB stages 4–6 plus severe AD-type pathology [92, 174], which may act synergistically [9, 27, 35, 172–174, 184, 185], influencing clinical features including a shorter duration or a more malignant course [169, 172]. AD neuropathology seems to be a more specific correlate of dementia than cortical αSyn pathology [169, 173]. Substantia nigra cell loss is more severe in PDD than in DLB [15], consistent with more advanced parkinsonism.

Multiple neurotransmitter deficits occur in PDD [29, 172], including loss of limbic and cortically projecting dopaminergic neurons in the mesocortical limbic system [172] and involvement of the cholinergic system with loss of neurons in the nucleus basalis of Meynert leading to cortical cholinergic denervation [9, 171, 186]. Severe pathology also involves the noradrenergic locus coeruleus, causing dysfunction of the related circuitry [170]. Pedunculopontine cholinergic cell loss occurs in hallucinating PDD patients but not in DLB, which may indicate a different pattern of degeneration of cholinergic input structures [187].

DLB is featured by the co-occurrence of Lewy/αSyn pathology involving cortical and limbic areas (Braak LB stages 3–6) and AD-related pathologies. While some authors suggest that high cortical LB burden is the only independent predictor of dementia in DLB [177], others consider AD-related pathology to be more important [188]; however, studies have shown a strong correlation between both cortical pathologies [169, 173].

The DLB clinical syndrome is positively correlated to the extent of LB pathology (LBP) and negatively to the severity of neuritic AD pathology, while Aβ load has no effect [189]. A subgroup with the clinical picture of DLB was shown to have minimal cerebral amyloid deposition [190]. The higher cortical LB load in the temporal and parietal regions, which seems to be a distinguishing feature of DLB, may account for the shorter latency to dementia and could be accelerated by the *APOE ε4* allele [177]. Further, αSyn is an important predictor of disease duration both independently and synergistically with tau and Aβ load [191].

Other co-occurring pathologies (cerebrovascular lesions, cerebral amyloid angiopathy, hippocampal sclerosis, argyrophilic grain disease, and TDP-43 deposits) in PDD (19%) and DLB-AD (31.3%) brains appear to be of minor importance [35, 172, 192–194], although they may influence the development of dementia [195]. Cerebrovascular lesions in DLB are relatively mild, showing an inverse relationship with the severity of LBP [196–198]. Cerebral microbleeds are more frequent in DLB than in PDD [199], with highest densities in the occipital lobe [200], but they appear to be independent of cerebral amyloid angiopathy [201].

#### Morphological overlap

Both PDD and DLB may show similar neuropathological features, with a variable mixture of αSyn/LB and AD-related pathologies (Table 4). A common pathophysiological factor is synaptic dysfunction due to the initial aggregation of αSyn in the presynapses causing functional disconnection [202] due to interference with axonal transport and neurotransmitter deprivation [178, 180, 203, 204]. The relationship between phosphorylated αSyn and tau accumulation to Aβ deposition in the cerebral cortex [205, 206] suggests that there is an overlap in the pathology between AD and DLB, and that Aβ promotes the accumulation of both αSyn and tau [35–37]. Thus, cognitive decline and related symptoms are not a consequence of αSyn-induced neurodegeneration alone since Aβ and tau pathologies also contribute to the overall deficits [33, 35–37, 207].

**Table 4:** Morphological overlap and dissimilarities between dementia with Lewy bodies (DLB) and Parkinson disease-dementia (PDD)

Morphological overlap	Morphological dissimilarities
Variable mixture of cortical and subcortical LB/αSyn pathology and AD-related pathology Similar Braak LB stages (4-6) and neuritic stages (5 or 6) Relationship between pαSyn and tau aggregation to Aβ deposition in frontal and temporal cortex Initial αSyn aggregation in pre-synapses inducing neurodegeneration via interference with axonal transport Postsynaptic protein downregulation	Higher Aβ load in cortex and striatum in DLB Neuritic plaque scores higher in DLB Higher cortical LB load in temporal and parietal cortex in DLB Increased tau loads in cortex and striatum in DLB More frequent and severe αSyn load in hippocampal subareas C2(3) in DLB Minor deviations in lesion pattern in SNc Pedunculopontine cholinergic cell loss in hallucinating PDD, but not in DLB Higher 5-HT1A receptor binding in cerebral cortex in DLB More frequent cerebral microbleeds in DLB

*LB* Lewy body, *AD* Alzheimer disease, *SNc* substantia nigra pars compacta

#### Morphological differences

Despite many similarities, several morphological differences have been demonstrated, including higher Aβ load in striatum [34, 208], cortex, and claustrum [33, 177, 197, 209–211] and in the entorhinal cortex, amygdala, and putamen in DLB [27]. The presence of Aβ in DLB and less so in PDD, along with its great sensitivity to differentiate between the disorders, have been extensively investigated [33, 34, 177, 209], with a hierarchy PD < PDD < DLB in both Aβ and tau burden [143] (Table 4).

Further differences include a more severe αSyn load in hippocampal subarea C2 in DLB [29] and in amygdala in DLB compared to in PDD (78.7% vs. 36% and 92% vs. 30%, respectively) [212], whereas αSyn loads in PD are highest in the cingulate cortex [33]. Other deviations include the severity and distribution pattern of lesions in substantia nigra pars compacta (predominant neuronal loss in the ventrolateral parts in PDD versus more severe damage in the dorsolateral parts in DLB) and less marked nigral neuronal loss causing less severe postsynaptic dopaminergic upregulation [209, 213]. Additionally, significantly higher 5-HT1A receptor binding density in the cortex was seen in DLB compared to PDD [214]. The heterogeneous neurochemistry of both DLB and PDD, which depends on differences in pathology, suggests that these αSyn-related disorders and AD share a common, underlying molecular pathogenesis; however, this needs further elucidation.

### Pathogenic aspects

The clinicopathological features of DLB, PDD, and other synucleinopathies are highly variable and heterogeneous [9, 29, 215–217], although the spread of LBP was originally suggested to be uniformly ordered according to the Braak scheme [218, 219]. There are three current major staging systems in use for LB disorders, including one for PD [218, 219], one for DLB [23], and revised guidelines for LB disease [2, 220, 221]. Based on semiquantitative assessment of LBs in large autopsy series, a staging of the chronological spread of LBP was proposed to designate its predictable caudo-rostral sequence in the CNS, which, however, is not identical with the spreading and location of αSyn pathology [222, 223]. Cases with severe LBP (Braak ‘neocortical’ stages 5 and 6) that show overlap or transition between PD and DLB are frequently associated with cognitive impairment, which increases with progressing neuropathological changes [223].

The validity of the Braak staging scheme, which corresponds roughly to the classification of LB disorders as either a (1) predominantly brainstem pathology, (2) limbic system (limbic/transitional type) pathology, or (3) diffuse neocortical pathology [224], has gained wide support as a standard for assessment of LBDs [98, 225, 226], but has also been a matter of vigorous debate [216, 227–231]. The Braak staging scheme often, but not consistently, shows acceptable correlations between morphological findings and clinical data, mainly in a subgroup with early onset and prolonged disease duration [232], whereas a new unified staging system allows the classification of all cases of LBDs, including PD, PDD, DLB, incidental LBD, and DLB-AD [220].

According to the Braak scheme, αSyn aggregates, forming the major components of LBs, and Lewy neurites appear first in the olfactory structures and enteric nervous system and then progressively spread into the brain, moving from cell to cell (neuron to neuron) and through neuronal circuits in a ‘prion-like’ manner, thus contributing to synaptic failure [233] due to impaired axonal transport and accounting for the progression of LBP [234–236]. More recently, it has been hypothesized that αSyn itself may be a critical factor in mediating transmission of disease pathology by such a ‘prion-like’ process, which appears essential for the pathogenesis of both PDD and DLB [237]. It remains to be seen if the species of aggregates of αSyn responsible for propagation and neurodegeneration are different and whether the various strains of αSyn fibrils underlie the differences in cellular and regional distribution of lesions in different synucleinopathies, as has been observed following the injection of αSyn aggregates in animal models [238, 239].

An essential problem in distinguishing between DLB and PDD is the impact of AD-related pathology and its co-occurrence with LBP, although both types of lesion have been shown to be strongly correlated with one another [169, 173]. However, recent clinicopathological studies showed that the clinical features of DLB are the consequence of multiple regional pathologies that are less pronounced in PDD [9, 27, 30, 73]. Nevertheless, the genetic and molecular mechanisms responsible for the, at least partially, different pathogenetic factors of both disorders await further elucidation.

### Therapy

Currently, there are no disease-modifying therapies for LBDs available (however, see [240]), although robust evidence supports the use of cholinesterase inhibitors (ChEIs) to treat these disorders [241, 242], related to the reduction of cholinergic markers in both PDD and DLB [243, 244]. Meta-analyses have indicated beneficial effects of both donepezil and rivastigmine for cognitive and psychiatric symptoms in both disorders [245–248], while only one study found an effect of memantine in PDD [249]. The efficacy of memantine in DLB is thus less clear, but may have benefits either as monotherapy or as adjunctive to a ChEI [241]; further, it induced longer survival in patients with DLB and PDD [250]. Although the effects were relatively small, ChEIs gave a better response of cognitive impairment in DLB and PDD than in AD [251], and may produce reduction in apathy, visual hallucinations, and delusions [252]. The use of antipsychotics should be avoided given the risk of serious reactions in DLB [2, 253]. When atypical antipsychotic agents are needed, quetiapine, and particularly clozapine, are less likely exacerbate parkinsonism [251]. Levodopa is generally well tolerated, but produces significantly less motor response in DLB than in PD and may be associated with an increased risk of psychosis [242, 254, 255]. Additionally, strategies to decrease the level of αSyn, to prevent cell-to-cell transmission of misfolded αSyn, and deep brain stimulation of the cholinergic nucleus basalis of Meynert have been discussed [39, 256]. Future therapeutic strategies should include disease-modifying strategies, possibly based on recent vaccination trials against αSyn, Aβ, and tau proteins [257, 258]. Preliminary results of anti-αSyn-immunotherapy in a combined model of synucleinopathy [259] may open the way to potential new treatments. A recent review of non-pharmacological interactions for DLB gave no definite results [260], while bilateral deep brain stimulation of the NBM for PDD showed potential improvement of neuropsychiatric symptoms [261].

## Conclusions

DLB and PDD are major neurocognitive disorders with LBD, sharing many clinical, genetic, pathophysiological, imaging, and morphological features. Thus far, a clear and objective distinction between the two entities, other than the arbitrary timing of the appearance of cognitive and motor impairments (1-year rule), has not been established [5, 10, 15, 220], while others maintain that the two entities may merge [262] or may become the same disease [17]. The revised Movement Disorder Society clinical definition of PD, considering DLB with presence of parkinsonism a ‘DLB subtype of PD’ [18, 31], was criticized since it would confuse rather than clarify the distinction between both entities [3]. However, the 1-year time period may not be the optimal method for diagnostic distinction between both disorders [3] since cognitive decline has been reported to start as early as 6 years prior to PD diagnosis [263]. Yet, it appears questionable whether this and other recent clinical studies on impaired cognition years before manifestation of parkinsonism [264] may blur the distinction between PD and DLB, which has been supported by recent neuroimaging and postmortem studies indicating that, in addition to predominant LB/αSyn pathology, AD-related lesions may contribute to the timing of dementia onset relative to motor signs [177].

The clinical pictures of both phenotypes, characterized by recent diagnostic criteria (for DLB [2] and for PDD [24, 72, 84]), despite individual variability, show many overlapping and distinguishing features [3, 8, 126, 265] (Table 1).

Several genetic markers have been shown to be risk factors for DLB and/or PDD, with some differences among them (Table 3). However, it appears premature to recommend genetic testing for clinical diagnosis and differentiation between DLB and PDD. A number of indicative and supportive biomarkers may contribute to the clinical diagnosis of probable DLB and PDD (Table 2).

Despite considerable overlap between DLB and PDD, recent neuroimaging and postmortem studies have demonstrated differences in the quantity and distribution pattern of LB/αSyn and AD-related pathologies between these two entities (Table 4). A correlation between these lesions suggests (1) a synergistic/additive or triggering effect between these protein pathologies [266], with increasing levels of AD pathology inducing an increasing burden of αSyn pathology; (2) an overlap in the pathology between DLB and AD; and (3) that the cognitive decline and related symptoms are not a consequence of αSyn-induced neurodegeneration alone, but of mixed pathologies contributing to the overall deficits [30, 35, 37, 183, 207, 266].

A possible interpretation of the available data would be that PDD and DLB are sub/phenotypes or two ends of the LBD spectrum [19], in which DLB may reside at the more severe side next to AD, while incidental LBD would be on the other (initiating) end [267]. The suggested spectrum is as follows: incidental LBD > PD/non-demented > PDD > DLB > DLB/AD nearing AD. Recent GWAS studies suggested, as another possibility, that DLB and PDD would be distinct diseases with shared genetic risk features with PD and AD [32]. Although some genetic factors that predispose to the development of dementia may differ in PDD and DLB, further extensive GWAS studies in autopsy-confirmed cohorts are warranted.

### Future perspectives

DLB and PDD are clinically similar illnesses, distinguished on the basis of the relative timing of dementia and parkinsonism (the 1-year rule). In view of the heterogeneity of the clinical course and symptomatology of both disorders that share the same pathophysiology [30], the question of whether this is a biologically valid distinction, or whether they are merely subtypes in a continuum of LBDs remains to be elucidated based on the results of combined biomarkers, new molecular imaging tracers [268, 269], and multimodal imaging [106]. Their distinction would be useful for further diagnostics and, in particular, new and disease-specific preventive and curative measurements.

At present, neuropathological (differential) diagnosis of DLB and PDD with no or insufficient clinical data would be difficult [181]. However, according to the preliminary criteria proposed in Table 5 (which need further validation and reproducibility), this may be possible. In view of the recent data on the clinical diagnostic criteria for DLB [68], their accuracy remains limited, while, to the best of our knowledge, no comparable studies are available for PDD. In order to support the notion that DLB and PDD are separate diseases, a unique pathogenic process should be identified for either one or the other. Therefore, at present, they cannot be strictly separated as distinct, whereas clinical, imaging, and morphological parameters can distinguish DLB from AD and frontotemporal dementia. The solution of this problem – if at all possible – warrants extensive multidisciplinary studies designed to shed further light on the relationship between PDD and DLB, including identifying genetic and environmental risk factors, and improving our understanding of the biological mechanisms responsible for their pathogenesis such that preventative or curative management can be developed [270].

**Table 5:** Preliminary neuropathological features of dementia with Lewy bodies (DLB) and Parkinson disease with dementia (PDD)

Type of lesion	DLB	PDD
LB / αSyn pathology	Both subtypes are characteristic by a combination of progressed LB pathology (LB Braak stage 5–6) and AD pathology of variable severity and extent	
Aβ load	More severe and extended in cortex and striatum	Less severe and less extended
Tau load	Higher tau load, particularly in medial temporal cortex	Comparatively low tau load in cortex and striatum
αSyn load (hippocampus)	CA 1/2 more severely involved	CA 2/3 more frequently involved
SN neuronal cell loss	Preferentially involving dorsolateral substantia nigra pars compacta	More severe, preferentially involving medioventral SNc
Pedunculopontine cholinergic cell loss	Negative	Positive in hallucinating PDD
5-HT1A receptor binding density in cortex	Higher	Lower
Cortical LB load	Higher in temporal & parietal cortex, hippocampus	Diffuse or focal

*LB* Lewy body, *AD* Alzheimer disease, *SN* substantia nigra

Nevertheless, the wide acceptance of the term DLB is evidence of its clinical utility, which is likely to result in the maintenance of the term; it is useful in the differential diagnosis of cases presenting with cognitive decline. Whether such patients are likely to develop extrapyramidal symptoms (DLB) or not (AD, etc.) has prognostic value and indicates the type of therapy (e.g., typical or atypical neuroleptics) and is thus of clinical importance. Although we favor the concept of a continuum between DLB and PDD, it must be recognized that biological factors must exist that determine whether the synucleinopathy will present earlier with cognitive decline or with extrapyramidal features. Identifying such factors is important scientifically and may lead to the development of disease-modifying therapies.
